# Multidisciplinary Management of Acute Tetraparesis in an Infant with Achondroplasia, with a Focus on Anesthetic Strategies: A Case Report

**DOI:** 10.3390/children12020164

**Published:** 2025-01-29

**Authors:** Barbora Nedomová, Robert Chrenko, Salome Jakešová, Petra Zahradníková, Martin Hanko, Ľubica Tichá

**Affiliations:** 1 Department of Pediatric Anesthesiology and Intensive Care, Faculty of Medicine, Comenius University, Faculty of Medicine, Slovak Medical University and National Institute of Children’s Diseases, 833 40 Bratislava, Slovakia; 2 Faculty of Medicine, Slovak Medical University, 831 01 Bratislava, Slovakia; 3 Department of Pediatric Neurosurgery, National Institute of Children’s Diseases, 833 40 Bratislava, Slovakia; 4 Department of Radiology, National Institute of Children’s Diseases, 833 40 Bratislava, Slovakia; 5 Department of Pediatric Surgery, Faculty of Medicine, Comenius University and National Institute of Children’s Diseases, 833 40 Bratislava, Slovakia; 6 Department of Neurological and Spinal Surgery, Penta Hospitals Bory, 841 03 Bratislava, Slovakia; 7 Department of Pediatrics, Faculty of Medicine, Comenius University and National Institute of Children’s Diseases, 833 40 Bratislava, Slovakia

**Keywords:** achondroplasia, infant, anesthesia, foramen magnum stenosis, cervical injury

## Abstract

Background/Objectives: This report details a rare instance of an infant with achondroplasia developing acute tetraparesis after a cervical whiplash injury, highlighting key multidisciplinary management considerations and specific anesthetic strategies to mitigate potential risks. Case presentation: A 1-year-old boy with achondroplasia presented with acute tetraparesis after a whiplash injury. Initial craniocervical computed tomography demonstrated a reduced volume of the posterior fossa, foramen magnum stenosis, and ventriculomegaly, without any fractures or dislocations. Moreover, magnetic resonance imaging (MRI) revealed pathological signal changes in the medulla oblongata, cervical spinal cord in segments C1 and C2, and the posterior atlantoaxial ligament. After initial conservative therapy and head immobilization using a soft cervical collar, partial remission of the tetraparesis was achieved. Two weeks post-injury, microsurgical posterior fossa decompression extending to the foramen magnum and C1 laminectomy was performed under general anesthesia with intraoperative neuromonitoring. Following an unsuccessful intubation attempt using a fiberoptic bronchoscope, successful airway management was achieved using a combined technique incorporating video laryngoscopy. Venous access was secured under ultrasound guidance. The patient exhibited complete remission of neurological symptoms by the third postoperative month during follow-up. Conclusions: This case report underscores the crucial need for a multidisciplinary approach in managing children with achondroplasia, especially with foramen magnum stenosis and complex cervical spine injuries. Anesthetic management required meticulously planned airway strategies using advanced techniques like video laryngoscopy and fiberoptic bronchoscopy to reduce airway risks. It also highlights the importance of conservative therapy paired with timely neurosurgical intervention, resulting in the patient’s full recovery.

## 1. Introduction

Achondroplasia is a rare genetic bone disorder that causes a disproportionately short stature. Affected individuals have a rhizomelic shortening of the limbs, macrocephaly, and characteristic facial features with frontal bossing and midface retrusion [1]. In addition, it is associated with possible foramen magnum stenosis, increased rates of obstructive and central sleep apnea, and a significant risk of morbidity and sudden death in infants [1,2,3,4]. The mortality rate due to foramen magnum stenosis in children with achondroplasia varies among reports, ranging from 2–7.5% [5,6,7].

Pediatric anesthesiologists face challenges in the administration of anesthesia due to the specific anatomical and physiological characteristics of children with achondroplasia [8,9,10]. Achondroplasia is characterized by a difficult airway and spinal manifestations, including maxillary hypoplasia, upper airway stenosis, tracheobronchomalacia, and scoliosis [8]. Proper airway management, correct patient positioning, safe intravenous access, and appropriate dosing of anesthetic agents are special considerations when planning anesthesia for children with achondroplasia, to ensure a safe surgical procedure and prevent catastrophic iatrogenic events during anesthesia, such as traumatic spinal cord injury or atlantoaxial dislocation.

This report describes the case of a child with achondroplasia who developed neurological deficits after a cervical whiplash injury. This case report highlights the critical importance of a multidisciplinary approach for managing children with achondroplasia, particularly in the presence of foramen magnum stenosis and complex cervical spine injuries.

## 2. Case Presentation

**Figure 1:**
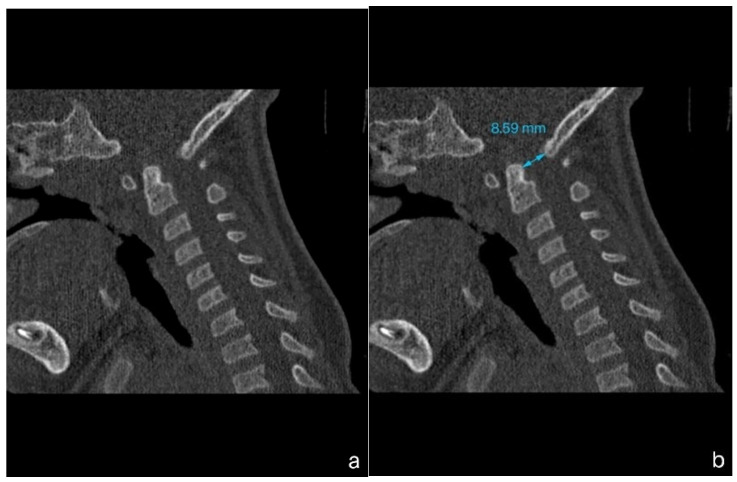
Computed tomography (CT) imaging of a child with achondroplasia following a whiplash injury. (**a**) Sagittal plane bone window CT scan demonstrating stenosis at the craniocervical junction, with no fractures or dislocations present. (**b**) Sagittal plane bone window CT scan highlighting a reduced dens-opisthion distance, with the anteroposterior diameter at the C2–C0 level measuring 8.5 mm.

**Figure 2:**
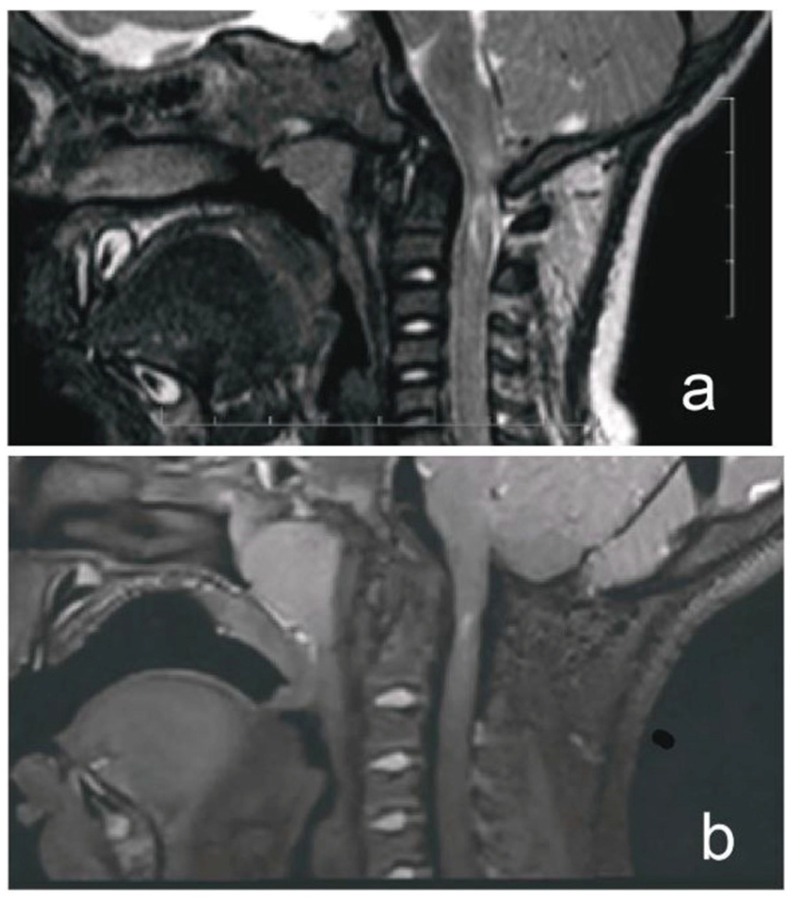
MRI of a child with achondroplasia who sustained a whiplash injury. (**a**) T2-weighted magnetic resonance image (MRI) of a 1-year-old boy with achondroplasia who developed acute tetraparesis (Medical Research Council grade: 1/5 in the upper extremities, 2–3/5 in the lower extremities) after a whiplash injury, showing foramen magnum stenosis, pathological signal changes in the medulla oblongata, cervical cord in segments C1 and C2, and posterior atlantoaxial ligament. (**b**) T2-weighted sampling perfection with application of optimized contrast using different flip angle evolution MRI obtained at our hospital 4 years postoperatively demonstrated the disappearance of pathological signal change in the medulla oblongata, a residual area of central hyperintensity in the C1 segment of the cervical cord, as well as a reduced anteroposterior diameter of the cord at this level. There was also a perimedullary flow void signal in the cervicocranial junction, indicating cerebrospinal fluid (CSF) flow.

A 1-year-old boy with achondroplasia, measuring 71 cm in height and 8400 g in weight, experienced a whiplash injury. The mechanism of injury was described as follows: “After the mother threw the child up in the air and caught him, his head jerked violently, following which he became motionless”. The patient was regularly monitored for achondroplasia at another medical center without any mention of foramen magnum stenosis. Upon admission, the patient presented with signs of achondroplasia, including macrocephaly, a depressed nasal bridge, and a large fontanel measuring 2 × 2 cm. At birth, the patient’s head circumference was 39 cm, and it was 51 cm at the time of admission. A neurological examination revealed acute flaccid tetraparesis with a Medical Research Council (MRC) grade 1/5 in the upper extremities and MRC grades 2–3 in the lower extremities. No other neurological deficits were observed. Initial craniocervical computed tomography demonstrated a reduced volume of the posterior fossa, foramen magnum stenosis, and ventriculomegaly, without any fractures or dislocations (Figure 1a,b). However, magnetic resonance imaging (MRI) revealed foramen magnum stenosis and pathological signal changes in the medulla oblongata, cervical spinal cord in segments C1 and C2, and the posterior atlantoaxial ligament. Additionally, morphological changes in the skull base and basilar impressions were observed (Figure 2a). The patient was monitored in the PICU and experienced an episode of hypoxemia, with an oxygen saturation of 79% on the first night after the injury. This episode resolved spontaneously after administering oxygen through a face mask for 10 min at a rate of 3 L/min. The patient remained respiratory and hemodynamically stable and did not experience any complications. He was breastfed and exhibited no swallowing disorders. Symptomatic treatment included regular analgesia with metamizole, supplemented by rectally administered paracetamol and the use of a soft cervical collar. The neurosurgeon recommended delaying the surgical therapy based on established principles to minimize the risk of intraoperative lesioning of compressed neural structures during the acute phase of the injury and to allow sufficient time for the edema-related compression of neural tissues to subside. A gradual remission of neurological symptoms was observed, and a microsurgical decompression was finally performed two weeks after the injury, after the flaccid tetraparesis subsided gradually to a MRC grade of 3/5 for the upper extremities and to an MRC grade of 4/5 for the lower extremities.

Preoperative evaluation revealed an American Society of Anesthesiologists grade of 3. The patient arrived in the operating room with a 24 G peripheral vein catheter secured on the dorsum of the left foot. Upon arrival, electrocardiographic and near-infrared spectroscopic monitoring were initiated, along with measurements of peripheral oxygen saturation, noninvasive arterial pressure, and body temperature prior to anesthesia induction. The preoperative hemoglobin level was 10.8 g/dL, and the hematocrit was 32.8%, with blood test results showing no abnormalities.

Given the diagnosis of achondroplasia and a history of cervical trauma, fiber optic-guided nasal intubation was selected to minimize manipulation of the cervical spine during airway management. This approach was chosen based on the anatomical and clinical specifics of the patient, emphasizing a safe and controlled method for securing the airway. Maintaining spontaneous respiratory activity was crucial to reducing the risk of cervical instability and related complications. Video laryngoscopy was prepared as a backup to address potential challenges, including limited visualization or failure of the primary plan.

Manual inline stabilization of the head and neck was provided by the assistant. The patient was drowsy but stable. Intravenous administration of 1% propofol at a dose of 10 mg, along with 0.5 mL (2.5 µg) of intravenous sufentanil, was performed. Inhalational anesthesia was initiated with 6% sevoflurane in 70% oxygen with air (fresh gas flow of 6 L/min) via a face mask. After achieving an adequate depth of anesthesia, approximately 3 min later, the sevoflurane concentration was reduced to 4% to maintain sufficient anesthesia during Plan A. The depth of anesthesia was objectively monitored based on the presence of miosis, absence of ocular activity, and absence of a motor response to a strong anterior displacement of the mandible. The patient demonstrated adequate spontaneous ventilation.

The initial airway assessment revealed limited mandibular protrusion and macroglossia, increasing the risk of difficulties in securing the airway. Based on these findings, the primary plan (Plan A) involved fiberoptic-guided nasal intubation. A lubricated, flexible fiberoptic bronchoscope, the Ambu^®^ aScope™ 5 Broncho (Ballerup, Denmark), with an outer diameter of 2.7 mm, was used in conjunction with a 3.5 mm cuffed endotracheal tube (ETT), which was successfully inserted through the right nostril. The 3.5 mm cuffed ETT was selected due to the risk of smaller airway dimensions commonly associated with achondroplasia, as well as the patient’s weight (8.5 kg). During the first attempt, a Cormack–Lehane Grade 3 view was observed, and the intubation attempt was unsuccessful due to significant anatomical challenges. Prior to the second attempt, a bolus of 10 mg of propofol was administered intravenously, slowly and cautiously, to deepen anesthesia and preserve spontaneous respiratory activity. Despite this adjustment, the second attempt was also unsuccessful, with persistent difficulties in visualizing the vocal cords due to macroglossia and excessive secretions intermittently obscuring the view. No additional bolus of anesthetic was administered before the third attempt; however, the visualization of the vocal cords remained unsuccessful, and the intubation attempt failed. After three unsuccessful attempts under Plan A, the decision was made to transition to Plan B. Throughout Plan A, peripheral oxygen saturation (SpO_2_) was maintained between 94% and 98%.

Plan B involved the use of a Karl Storz C-MAC videolaryngoscope (Karl Storz, Germany) with a size 2 Miller blade, which provided better tongue displacement and enhanced access to the glottis. The administration of 4% sevoflurane in 70% oxygen with air was continued through a face mask with a fresh gas flow of 6 L/min. After deciding to proceed with Plan B, a bolus of 20 mg of 1% propofol was administered, and ventilation was supported with gentle manual positive pressure ventilation. Following adequate preoxygenation, the videolaryngoscope was inserted during an apneic pause, providing a clear Grade 1 view. However, limited oral cavity space caused by macroglossia prevented direct insertion of the endotracheal tube. After visualizing the glottis, an additional bolus of 10 mg of propofol was administered, and the fiberoptic bronchoscope was used to guide the endotracheal tube into the trachea. The ETT cuff was inflated to 20 cm H_2_O under controlled conditions, ensuring the airway was securely established.

Peripheral oxygen saturation (SpO_2_) was continuously monitored throughout the airway management process. During Plan B, SpO_2_ ranged from 92% to 95%, with no significant episodes of desaturation observed.

After successful intubation, the sevoflurane concentration was reduced to 3%, and anesthesia was continued in combination with 40% oxygen with air at a fresh gas flow of 2 L/min. The combination of fiberoptic bronchoscopy and video laryngoscopy proved critical in managing this difficult airway while minimizing potential trauma.

Following the insertion of two additional peripheral venous cannulas (24 G on the dorsum of the right hand and 22 G in the antecubital fossa of the left upper limb under ultrasound guidance), anesthesia was transitioned to total intravenous anesthesia using a combination of propofol and remifentanil. This approach ensured stable anesthesia and optimal analgesia throughout the procedure.

Subsequently, a 6 Fr permanent urinary catheter was inserted for diuresis monitoring.

After the preoperative positioning of the patient to the prone position and prior to draping the surgical field, the patient’s blood pressure was within acceptable limits, with systolic pressures ranging between 92–94 mmHg and mean arterial pressures (MAP) between 60–62 mmHg. During draping, a decrease in systemic blood pressure was observed, with systolic blood pressure dropping to 70 mmHg and MAP to 45 mmHg, indicative of significant hypotension and insufficient perfusion pressure. This decrease in blood pressure was likely caused by relative hypovolemia due to vasodilation associated with anesthesia. This effect was further compounded by the lack of active patient manipulation during draping, despite adjustments to the depth of anesthesia. To address the hypotension, an initial bolus of 50 mL of Plasma-Lyte was administered, followed by a continuous infusion of crystalloids. When blood pressure did not improve, a bolus of 100 mL of 5% albumin was administered. Despite these interventions, blood pressure did not return to adequate levels, necessitating the initiation of a norepinephrine infusion at a dose of 0.05 µg/kg/min, which was subsequently titrated to a maximum of 0.1 µg/kg/min to achieve and maintain hemodynamic stability. The patient was operated on in the Concorde prone position, with the head lying on a headrest. Electrodes for intraoperative neuromonitoring (IONM) were placed, and somatosensory evoked potentials (SSEP), transcranial motor evoked potentials (tcMEP), and brainstem evoked potentials (BAEP) were monitored. A midline incision from the inion to the C2 spinous process was made, and posterior fossa decompression, foramen magnum decompression, and C1 laminectomy were performed. After bony and ligamentous decompression of the cervicocranial junction, intraoperative ultrasonography with a B-probe demonstrated a perimedullary CSF signal of more than 2 mm between the posterior cord and dura, which was considered satisfactory cord decompression [11]. The IONM parameters were uneventful from the beginning until the end of the surgery. Multilayer wound closure was performed in a standard fashion. The surgery lasted 125 min under 225 min of general anesthesia.

Over the entire procedure, a total of 270 mL of crystalloids and 100 mL of 5% albumin were administered to compensate for a blood loss of 30 mL and to maintain intravascular volume. Perfusion was closely monitored using urine output, maintained at 1 mL/kg/h, capillary refill time, and mean arterial pressure, which normalized following norepinephrine administration. The patient remained normothermic throughout the procedure, with no episodes of arrhythmias or hemodynamic instability observed.

Following the discontinuation of propofol and remifentanil, the patient demonstrated adequate spontaneous ventilation, regular respiratory effort, and sufficient tidal volumes. Oxygen saturation (SpO_2_) was maintained above 95%, and end-tidal CO_2_ (EtCO_2_) was measured at 45 mmHg. Hemodynamic parameters remained stable, with no signs of increased airway secretions, respiratory distress, or significant airway edema.

Extubation was performed under deep anesthesia after confirming sufficient spontaneous respiratory activity, stable oxygenation, and the absence of contraindicating factors. The procedure was carried out smoothly under close monitoring to ensure airway patency and adequate ventilation.

The patient was extubated without complications and received post-operative analgesia with intravenous acetaminophen (10 mg/mL, 100 mg) 20 min prior to extubation, and intravenous tramadol (20 mg) before leaving the operating room. The procedure was successfully completed, and the patient was transferred to the pediatric intensive care unit for further care and vital sign monitoring to maintain stability.

The laboratory evaluation, conducted 2 h after the admission to the pediatric intensive care unit, revealed a hemoglobin level of 10.1 g/dL and a hematocrit of 29.6%. Coagulation parameters were normal, and the blood test results showed no abnormalities. Postoperative chest radiography did not reveal any pathological findings. MRI scans obtained on the first postoperative day showed adequate decompression of the foramen magnum with regression of the medullary signal changes.

**Figure 3:**
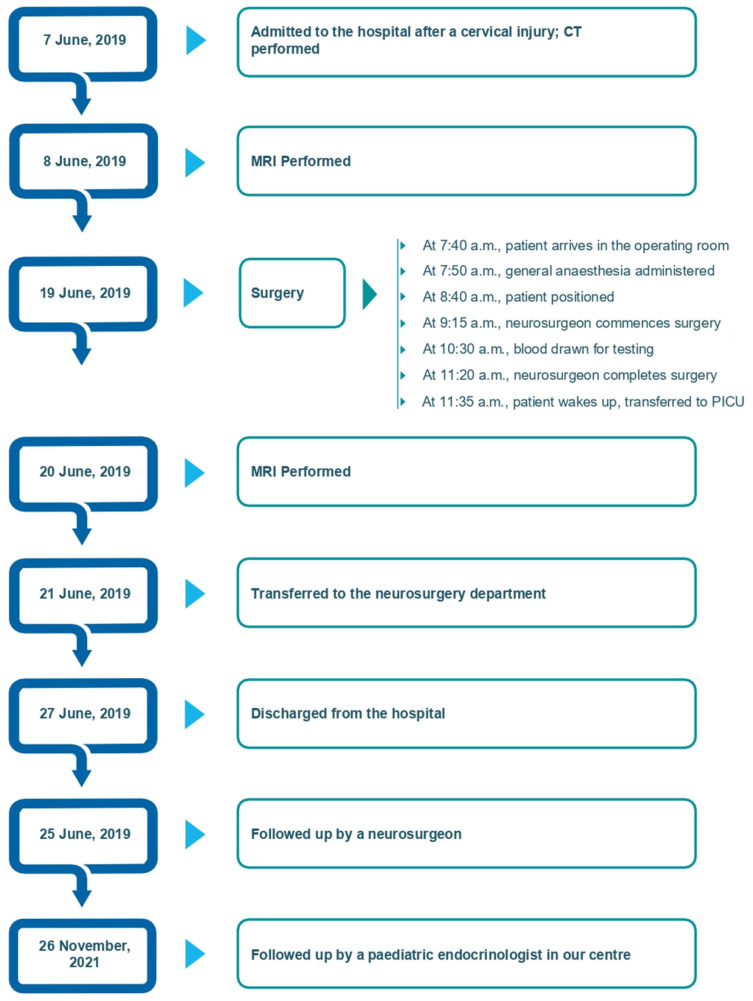
Timeline of the case.

The patient’s postoperative course was uneventful. The patient was transferred from the PICU to the neurosurgery ward 48 h post-operatively and was discharged from the hospital on day 8. After discharge, the patient wore a soft collar for one month and attended outpatient physiotherapy. Follow-up monitoring was conducted by a pediatric neurosurgeon at our hospital. Tetraparesis gradually improved, and no neurological symptoms were observed at outpatient follow-up 3 months postoperatively. Follow-up T2-weighted sampling perfection with the application of optimized contrast using different flip angle evolution MRI performed 4 years postoperatively revealed resolution of the edema, focal cervical cord atrophy, a small area of central myelomalacia at the C2 level, and a flow void signal, indicating adequate decompression of the craniocervical junction (Figure 2b). The patient then was checked-up on by a pediatric endocrinologist at our center. The timeline of the case is shown in Figure 3.

## 3. Discussion

Pediatric anesthesiologists face challenges in administering anesthesia to children with achondroplasia because their anatomical and physiological characteristics differ from those of children without achondroplasia. During mask ventilation and tracheal intubation, airway challenges may arise due to the features of achondroplasia, such as a large protruding forehead, midface hypoplasia, depressed nasal bridge, short maxilla, large mandible, small mouth opening, large tongue, narrowed nostrils, large adenoids, and a short neck, with limited head and neck movement [8,9,10]. Booth et al. reported that approximately 20% of children with achondroplasia suffer from obstructive sleep apnea, the most prevalent type of sleep-disorder-associated breathing [12]. Obstructive sleep apnea is caused by abnormal orofacial structures and factors commonly observed in children of average height, such as adenotonsillar hypertrophy and obesity.

Beyond airway considerations, children with achondroplasia frequently require neurosurgical procedures due to conditions like foramen magnum stenosis, spinal canal stenosis, and hydrocephalus. The most prevalent issue leading to neurosurgery is cervicomedullary compression caused by foramen magnum stenosis. The main reasons for surgical intervention include apnea or cyanosis (48%), changes in T2-weighted spinal cord signaling on MRI (15%), myelopathy (27%), and delays in reaching motor development milestones (15%) [13].

The standard procedure involves posterior fossa decompression (PFD), which targets the decompression of the foramen magnum. In approximately 65% of cases, cervical laminectomy—most frequently of C1—is also performed alongside the PFD. The decision to include duraplasty is typically individual, considering factors such as the presence of a syrinx or a continued narrow canal despite sufficient bony decompression [14]. We consider that the adequacy of decompression and the indication for additional duraplasty can be determined using intraoperative ultrasonography in an analog way as in patients with Chiari malformation [12,15]. In our case, after foramen magnum decompression and C1 laminectomy, we observed adequate CSF flow dorsal to the cervicomedullary junction (more than 2 mm between the cervicomedullary junction and dura) on intraoperative ultrasonography; therefore, additional duraplasty was not indicated.

Although high recovery rates and good outcomes are typical in patients with foramen magnum stenosis, the results refer mostly to patients who underwent elective surgical decompression [13].

The optimal management of patients with acute neurological injury resulting from a stenotic foramen magnum in achondroplasia has yet to be standardized in the literature. In our patient, surgical decompression was considered necessary; however, determining the timing of surgery proved to be a critical decision. Timely intervention may significantly impact neurological outcomes, making it essential to carefully weigh the risks and benefits of early versus delayed surgical intervention.

In our case, we adopted a strategy of delayed surgical decompression analogous to that applied in the management of patients with acute traumatic central cord syndrome in the context of cervical spinal canal stenosis. For patients with significant persistent cord compression, who consistently fail to progress after an initial period of improvement, surgery is indicated within 2–3 weeks following trauma without an arbitrary waiting period [16]. The rationale behind this approach is to avoid intraoperative lesioning of compressed neural structures in the acute phase of injury and to allow time for edema-related compression of neural tissues to subside.

From an anesthetic perspective, minimizing cervical movement during airway management is essential to prevent the exacerbation of cervicomedullary compression due to head and neck manipulation. Techniques should prioritize avoiding neck hyperextension and uncontrolled positioning. Airway management is particularly challenging when the degree of stenosis is unclear [1]. Difficulties in visualizing the larynx for tracheal intubation should be anticipated, and readily available airway adjuncts for difficult mask ventilation and video laryngoscopes should be used to avoid neck movement [1,9,10,17]. Anesthesiologists may encounter situations in which fiberoptic intubation is required instead of video laryngoscopy in children with difficult airways. Visualization of the larynx using a video laryngoscope can be challenging in children with achondroplasia due to limited mouth opening, mandibular hypoplasia, and syndromes associated with facial asymmetry [18]. However, as observed in our case report, securing the airway requires a combination of video laryngoscopy and fiberoptic intubation in some cases. In 2022, Dang et al. emphasized the importance of the awareness of the dilemmas associated with achondroplastic airways and perioperative considerations in adult patients [8]. Some recent studies have reported similar findings in children with achondroplasia.

In 2019, Ok et al. suggested, consistent with previous reports, that a smaller ETT might be required in children with achondroplasia because of the smaller dimensions of the airway compared with the standard estimated size based on age [3,8,10]. According to O’Donoghue et al., the size of the ETT should be appropriate for the patient’s age [10]. At our institution, when selecting an intubation cannula, we tend to select a smaller ETT than that intended for the child’s age.

Difficulties in obtaining intravenous access are common in children with achondroplasia, which can lead to delayed care. Ultrasound-guided peripheral intravenous cannulation has been shown to be a successful method for obtaining intravenous access in these children [1,19]. Additionally, alternative techniques, such as near-infrared imaging, have been shown to be valuable in locating veins for cannulation. Herein, ultrasonography was successfully performed for peripheral cannula insertion. According to O’Donoghue et al., the medication doses for children with achondroplasia should be calculated based on their ideal total or lean body weight, and special attention should be paid to the patient during surgery [10]. In our case, medication was administered according to the child’s body weight with a good effect.

Our report provides only sparse information on the medical management of the child prior to the trauma episode, as long-term management of this condition was not available in his country of origin. The child might have been spared a serious neurological deficit if foramen magnum stenosis had been diagnosed and surgically decompressed earlier. The surgery was timed to be performed after the myeloedema had resolved, mainly to prevent future myelin damage.

Appropriate management of the medical complications that may occur in children with achondroplasia is essential to achieve optimal outcomes and improve their quality of life [20,21]. The International Consensus Statement for Management of Patients with Achondroplasia 2022 recommends that families of children with achondroplasia should be informed and trained on their proper positioning and handling [1]. Preventive MRI screening at an early age in children with achondroplasia has previously been discussed [1,2,5,21].

## 4. Conclusions

This case highlights the critical importance of a multidisciplinary approach in managing children with achondroplasia, particularly in the presence of foramen magnum stenosis and complex cervical spine injuries. Early recognition of complications and routine imaging, including MRI, are essential in infants with achondroplasia. Anesthetic management should prioritize structured airway strategies utilizing advanced techniques such as video laryngoscopy and fiberoptic bronchoscopy to mitigate airway risks. The case emphasizes the necessity of a prepared backup plan in difficult airway management, as the failure of fiberoptic intubation (Plan A) highlighted the limitations of relying on a single technique. The combined use of video laryngoscopy and fiberoptic bronchoscopy proved effective in securing the airway with minimal trauma.

Timely and precisely planned neurosurgical intervention, guided by clinical and radiological findings, was crucial in preventing long-term neurological deficits. This case underscores the importance of individualized perioperative planning, specialized team coordination, and adherence to established guidelines to ensure safe and effective care.

## Data Availability

The datasets used and analyzed during the current study are available from the corresponding author upon reasonable request.
